# Financial Costs of the Zanzibar Elimination of Schistosomiasis Transmission Project

**DOI:** 10.4269/ajtmh.20-0252

**Published:** 2020-09-28

**Authors:** Paola Salari, Thomas Fürst, Stefanie Knopp, David Rollinson, Fatma Kabole, Mohammed I. Khamis, Mussa A. Omar, Oliver Bacon, Said M. Ali, Jürg Utzinger, Fabrizio Tediosi

**Affiliations:** 1 Swiss Tropical and Public Health Institute, Basel, Switzerland;; 2 University of Basel, Basel, Switzerland;; 3 Natural History Museum, London, United Kingdom;; 4 Neglected Diseases Program, Zanzibar Ministry of Health, Zanzibar, Tanzania;; 5 Public Health Laboratory-Ivo de Carneri, Chake Chake, Tanzania

## Abstract

We estimated the financial costs of different interventions against urogenital schistosomiasis, implemented by the Zanzibar Elimination of Schistosomiasis Transmission (ZEST) project, on Pemba and Unguja islands, Tanzania. We used available data on project activities, resources used, and costs reported in the accounting information systems of ZEST partners. The costs were estimated for all the activities related to snail control, behavior change interventions, the impact assessment surveys, and management of the whole program. Costs are presented in US$ for the full duration of the ZEST project from 2011/2012 to 2017. The total financial costs of implementing snail control activities over 5 years, excluding the costs for donated Bayluscide, were US$55,796 on Pemba and US$73,581 on Unguja, mainly driven by personnel costs. The total financial costs of implementing behavior change activities were US$109,165 on Pemba and US$155,828 on Unguja, with costs for personnel accounting for 47% on Pemba and 69% on Unguja. Costs of implementing biannual mass drug administration refer to the estimated 2.4 million treatments provided on Pemba over 4 years (2013–2016), and do not include the costs of donated praziquantel. The total cost per provided treatment was, on average, US$0.21. This study showed the value of exploiting administrative data to estimate costs of major global health interventions. It also provides an evidence base for financial costs and main cost drivers of implementing multiple combinations of intervention sets that inform decisions regarding the feasibility and affordability of implementing schistosomiasis control and elimination strategies.

## INTRODUCTION

Evidence regarding the financial implications of implementing schistosomiasis control and elimination interventions is important to inform program decisions on their feasibility and affordability for scaling up of activities.^1–3^ Yet, even large research programs usually do not consider including financial and economic analyses in their research and implementation plans.^4–7^ A recent systematic review of the cost of interventions to control schistosomiasis found large variations in the cost estimates and a low level of transparency in most of the studies.^8^ The review concluded that there is a pressing need for better-quality cost analyses. These conclusions are partially related to the lack of studies that embedded economic evaluations in implementation studies. Clearly, conducting these studies is time consuming, highly complex, and often requires accessing sensitive information that may highlight inefficient use, let alone misuse, of resources.^3,8^ There are, however, underused information and data available on the resources used in implementation research and in conducting disease control programs. The information is, for instance, comprised in budget data, financial reports, and activity reports by different stakeholders involved in implementing control interventions. This information can be used to conduct relatively basic but informative costing analyses that can be useful to assess the affordability of implementing such interventions.

The Zanzibar Elimination of Schistosomiasis Transmission (ZEST) project, implemented on the two main islands of the Zanzibar archipelago (i.e., Pemba and Unguja), from November 2011 to May 2017 is an example of a multiyear effort to eliminate schistosomiasis. Embedded in the ZEST project was a large-scale study of various interventions. In brief, a cluster-randomized trial—funded by the Schistosomiasis Consortium for Operational Research and Evaluation (SCORE)—associated with the ZEST project aimed to assess biannual mass drug administration (MDA) applied alone or in combination with either snail control or behavior change interventions for the reduction of *Schistosoma haematobium* infection prevalence and intensity in children from Zanzibar, and to compare the effect between these alternative approaches.^9^ At the end of the study in 2017, urogenital schistosomiasis was eliminated as public health problem from most of the 90 study sites.^9,10^ Moreover, the overall *S. haematobium* prevalence was significantly reduced from 6.1% to 1.7% in children aged 9–12 years and from 3.9% to 1.5% in adults aged 20–55 years from 2012 to 2017.^10^ However, 10 rounds of MDA with praziquantel over 5 years failed to interrupt *S. haematobium* transmission, even when supplemented by the implementation of additional elimination measures.^9,10^

In this study, we used the programmatic and financial information made available by the ZEST project partners to conduct a cost analysis aimed at estimating the financial costs of the interventions against urogenital schistosomiasis on the two main islands of Zanzibar. Experiences and lessons learned are important for managers of schistosomiasis and other neglected tropical disease (NTD) control programs.

## METHODS

### Study settings.

The two main islands of the Zanzibar archipelago, Pemba and Unguja, are located offshore the mainland of the United Republic of Tanzania in the Indian Ocean. The projected population of Zanzibar for 2019 was approximately 1.6 million inhabitants.^11^ The two islands are divided into 11 districts, which are subdivided into smaller administrative areas (i.e., shehias). According to the 2012 Tanzania Population and Housing Census, there were a total of 339 shehias: 212 on Unguja and 127 on Pemba. In 2018, there were 239 public primary schools and 102 private primary schools registered.^11^ The primary school net enrollment rate in the 2014/2015 household budget survey was estimated at 84.2%.^11^

### Zanzibar Elimination of Schistosomiasis Transmission (ZEST) project.

Urogenital schistosomiasis, caused by the parasitic blood fluke *S. haematobium*, used to be highly prevalent on the Zanzibar islands in the twentieth century.^12–16^ In the 1990s and early 2000s, large-scale preventive chemotherapy campaigns, where anthelmintic treatment was provided without prior diagnosis to large parts of the population, consolidated by socioeconomic growth and improved access to clean water, reduced the prevalence and intensity of infection, and hence, morbidity due to *S. haematobium* in the at-risk population.^17–19^ In 2011, the ZEST alliance was formed with the goal to eliminate schistosomiasis as a public health problem on Pemba and to interrupt *S. haematobium* transmission on Unguja over 5 years.^10,20^ The cluster-randomized trial within the ZEST project was implemented from November 2011 to May 2017. The trial was funded by SCORE via a major grant from the Bill & Melinda Gates Foundation.^21^ Box 1 shows the different partners involved in the ZEST project.

**Box 1:** Partners and stakeholders involved in the Zanzibar Elimination of Schistosomiasis Transmission project implemented from 2011/2012 to 2017

Institution/stakeholder	Location	Role
Neglected tropical disease control program of the Zanzibar Ministry of Health (ZNTD)	Unguja, United Republic of Tanzania	Local principal investigator; implementation of research activities and interventions on Unguja
Public Health Laboratory-Ivo de Carneri (PHL-IdC)	Pemba, United Republic of Tanzania	Local principal investigator; implementation of research activities and interventions on Pemba
Schistosomiasis Consortium for Operational Research and Evaluation (SCORE)	Athens, GA	Funder of the cluster-randomized trial, including annual parasitologic monitoring surveys, snail control, and behavior change interventions
Bill & Melinda Gates Foundation (BMGF)	Seattle, WA	Funder of SCORE at the University of Georgia
Natural History Museum (NHM)	London, United Kingdom	Study principal investigator; support of research on Unguja and Pemba
Swiss Tropical and Public Health Institute (Swiss TPH)	Basel, Switzerland	Study coprincipal investigator; support of research on Unguja and Pemba; responsible for cost analyses
Schistosomiasis Control Initiative (SCI)	London, United Kingdom	Implementation partner for biannual mass drug administration (MDA)
WHO	Geneva, Switzerland	Donation of praziquantel for biannual MDA
Bayer Crop Science AG (Bayer)	Monheim, Germany	Donation of Bayluscide for snail control

### Interventions of the ZEST project.

The three main ZEST interventions were MDA, snail control, and behavioral change.^9,10,20^ In brief, 10 rounds of MDA with praziquantel against urogenital schistosomiasis (plus albendazole or mebendazole against soil-transmitted helminthiases and sometimes also ivermectin against lymphatic filariasis) were implemented across the whole islands biannually from 2012 to 2016 as part of the routine activities of the NTD program of the Zanzibar Ministry of Health (ZNTD). An exception was the South district in Unguja, where, because of the absence of intermediate host snails and, hence, no *S. haematobium* transmission, there was no praziquantel distribution. With the exception of the sixth round on Pemba, each MDA round contained a community-wide treatment (CWT) approach.^9,10^ In CWT, trained community drug distributors (CDDs) were supposed to visit each household on the islands and, in a door-to-door approach, distribute the anthelmintic drugs to the eligible population. All individuals aged > 3 years were eligible to receive praziquantel (40 mg/kg administered by using a dose pole), with the exception of severely sick people and pregnant women. In the sixth round on Pemba, instead of the described CWT approach, health posts were installed, where people could visit and receive the drugs on request. In addition to CWT, to improve the coverage of the school-age population, school-based treatment (SBT) was implemented in five of the treatment rounds (nos. 4, 7, 8, 9, and 10) on both islands and on Pemba also in the sixth round. In SBT, children attending public primary schools, and from 2015, also nurseries, private, and religious schools (i.e., madrassas), received the anthelmintic drugs by their teachers and staff of the ZNTD by directly observed treatment. Treatment coverage data for all targeted shehias and schools were provided by the ZNTD through the registers of the CDDs and teachers, respectively.

For the implementation of the cluster-randomized trial, on each island, 45 shehias were randomized to one of three study arms: biannual MDA alone, biannual MDA plus snail control, or biannual MDA plus behavior change.

Snail control was implemented in 15 shehias per island, as part of the cluster-randomized trial from late 2012 until early 2017. Once the natural freshwater bodies in the study shehias were identified, they were regularly visited by trained snail control teams on the two islands. A visit included a snail survey at a specific site of the water body readily used by the local population for domestic and recreational activities (e.g., farming, fishing, bathing, playing, laundry, and washing dishes). For the snail survey, snail control team members searched the shoreline of the water body for snails, including the intermediate host snail of *S. haematobium*, which is *Bulinus globosus*. In case *B. globosus* were found, the snail control team sprayed the shoreline with a molluscicide (Bayluscide WP 83; Bayer Crop Sciences, Monheim, Germany) using a petrol sprayer or Hudson backpack sprayers to kill the snails. Snail control activities started in August 2012 and were implemented throughout the study years until February 2017, with exception of the rainy seasons. If no *B. globosus* were found, that area was not sprayed during that round. In Pemba, a total of 167 human–water contact sites were identified that were visited by the snail control team over the study period on a total of 556 days.^9,22^ At 71 among the 167 sites, *B. globosus* snails were found at least once, and 60 sites were treated with Bayluscide. On Unguja, a total of 121 human–water contact sites were identified that were visited by the snail control team over the study period on a total of 346 days.^22^ At 91 among the 121 sites, *B. globosus* snails were found at least once, and 65 sites were sprayed with Bayluscide.

In 15 randomly selected shehias per island, in addition to MDA, behavior change interventions were implemented. The interventions were developed in a participatory human-centered design approach, together with the local communities.^23–25^ Four main behavior change activities were carried out over time from late 2012 until early 2017: 1) classroom-based interactive teaching about schistosomiasis by school teachers who had received specific training by the staff of the behavior change teams on each island and were provided with a schistosomiasis tool kit containing teaching materials such as lesson plans, blood fluke picture, flip charts tailored to the local perceptions, and snail boards^23,24,26^; 2) school-based interactive “Kichocho days,” where the whole school was involved in games, dramas, and songs with health education messages about the prevention, control, and treatment of schistosomiasis; 3) community-based installment of 30 urinals on Pemba and 28 urinals on Unguja; and 4) community-based installment of 21 washing platforms on Pemba and 25 washing platforms on Unguja.^9^

### Parasitologic impact assessment surveys.

The impact of biannual MDA alone, or in combination with snail control or behavioral change interventions, was assessed in cross-sectional surveys conducted annually in 45 study schools and 45 shehias on Pemba and Unguja, respectively, as part of the cluster-randomized trial. Annually, from 2011/2012 to 2017, approximately 100 children from grades 3 and 4 (aged 9–12 years) in the primary schools and 50 adults (aged 20–55 years) living in the shehia communities of each of the 90 study sites were randomly selected and invited for participation in the study by experienced field-workers. Their urines were collected between 10 am and 2 pm and subsequently examined in the laboratory for *S. haematobium* eggs by single urine filtration microscopy of 10 mL of urine per participant and for blood in urine by reagent strips (Hemastix, Siemens Healthcare Diagnostics Ltd., Surrey, United Kingdom) by trained laboratory technicians.^27^

### Costing approach and data.

The study presented here is a retrospective cost analysis of the ZEST project that used available data on the project activities, resources used, and costs reported in the accounting information systems of multiple ZEST partners. To classify the costs, we first developed a reference cost model and a cost classification tool. The reference cost model was informed by the existing literature and exchanges with experts from the wider SCORE network. The reference cost model was structured into two hierarchical levels (i.e., macro- and meso-level) and used—to the extent possible—mutually exclusive and complete activity/process and associated expense/cost categories. For each category, be it at the higher or the lower hierarchical level, a short written definition and explanation was developed. Based on this reference cost model, a cost classification tool was established, which included all the details from the reference cost model and additional information considered relevant and useful for estimating the costs (e.g., reference/check/receipt/voucher number, information on partner, island, date of cost recording/entry or—preferable—true date of expenditure, allocation to the various intervention types/study arms, the reported currency and amount, and other potentially relevant information). All data obtained from the ZEST partners were classified according to the reference cost tool (see Supplemental Materials).

The cost analysis is based on the following budget and activity data: 1) Public Health Laboratory-Ivo de Carneri (PHL-IdC) data that included the costs and activities related to snail control, behavioral change interventions, parasitologic surveys, and the correlated management activities for the whole duration of the project on Pemba (2011–2017); 2) ZNTD data that included the costs and activities related to snail control, behavioral change interventions, parasitologic surveys, and the correlated management activities for the whole duration of the project on Unguja (2011–2017); 3) Schistosomiasis Control Initiative data that included the costs of the implementation of biannual MDA in Pemba between 2013 and 2016; and 4) Natural History Museum (NHM) data, which corresponded to procurements performed in the United Kingdom for equipment and material for the parasitologic and snail surveys on Pemba and Unguja, respectively, that were not available locally for the whole duration of the project. Whenever possible, for PHL-IdC, ZNTD, and NHM data, we assigned the costs to only one of the activities. When the costs referred to a generic management definition (e.g., general management expenses), we kept it as an extra single category. If the costs referred to two activities (e.g., snail control and behavior change, or snail control and parasitologic survey), we split the costs equally.

Following the same pattern for each of the main activities, the costs were assigned to four categories: 1) personnel, 2) equipment, 3) material, and 4) travel. Personnel costs depend on the income that a staff received and included the following subcategories: top-up for project involvement and allowances. As regards equipment costs, these included capital costs that were represented by means of transport (e.g., cars), microscopes, information technology (IT) infrastructures (e.g., computers, antivirus software, and printers), and other capital equipment that varied from building material to a simple bucket. Material costs refer to consumables goods, which were represented by laboratory material (e.g., gloves, microscopes slides, soap, and chemical substances) and office material (e.g., pencils, cartridges for printers, telephone, and internet vouchers). With regard to transport costs, these included gasoline and public transport (e.g., ferry or bus rides and longer-distance travels to attend management meetings). Of note, costs of gasoline in the PHL-IdC and ZNTD databases were not attributable to a specific activity. Hence, we summed up all the costs for gasoline related to any of the three activities (i.e., snail control, behavior change interventions, and parasitologic survey) carried out by the PHL-IdC and ZNTD and reallocated them to each of the three activities according to the days of work per month for each of them (namely, for the snail team: 5 days a week for 4 months in the years 2012–2014 and for 8 months in the years 2015–2017, for the behavior change team: 2.5 days per week for 12 months per year, and for the parasitology team: 5 days per week for 3 months per year).

The costs of praziquantel tablets for MDA and of Bayluscide for snail control were not included in our analysis because these were donated by the WHO and Bayer Crop Science, respectively. The costs of external experts that supported the implementation of the study were not included, as they were essentially related to the research activities but not to the program interventions.

The cost estimates of the activities related to snail control, behavior change, the parasitologic surveys, and for the management of the whole ZEST project are presented for the full duration of the project from 2011/2012 to 2017. The costs of the implementation of biannual MDA on Pemba are presented for the years 2013–2016 only. All costs are expressed in US$ and not adjusted for inflation, separately for Pemba and Unguja, and the following interventions and activities: snail control, behavior change, MDA, parasitologic surveys, and program management.

The costs of snail control are presented in terms of total costs, cost per day in the field, and cost per shehia covered. Of note, the costs of Bayluscide were not included, as this molluscicide was donated. The costs of behavior change interventions are presented in terms of total cost and cost per shehia covered. The costs of the parasitologic surveys are presented in terms of total costs, cost per shehia monitored, and cost per survey conducted, including both school surveys and community surveys. The costs of biannual MDA are presented in terms of total costs and cost per person treated. The cost of the anthelmintic drug praziquantel was not included, as the drug was donated. The costs attributed to managing the whole ZEST project locally in Zanzibar were presented only in terms of total costs, as these are costs attributed to all activity categories implemented on Pemba and Unguja.

## RESULTS

### Costs of snail control.

**Figure 1.**
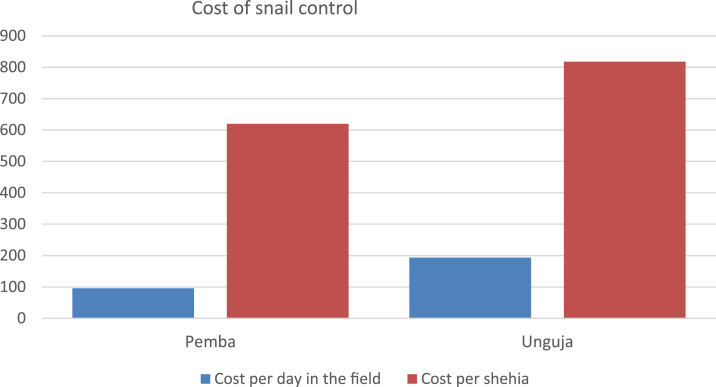
Snail control: cost per day in the field and cost per shehia covered—in US$. This figure appears in color at www.ajtmh.org.

**Figure 2.**
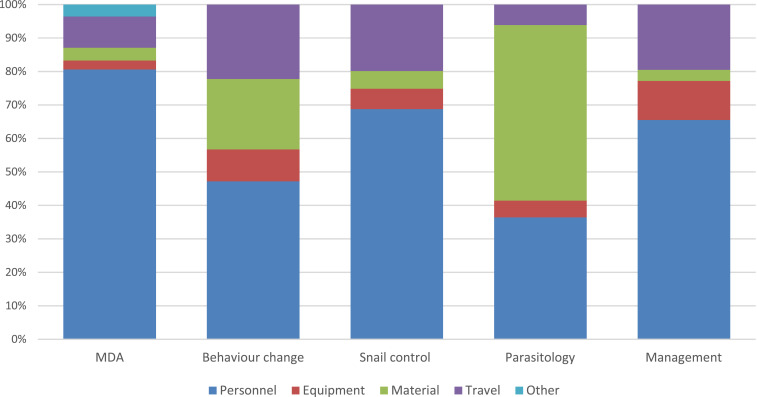
Cost structure by activities—total costs from 2012 to 2017 (2013–2016 for MDA) in Pemba. MDA = mass drug administration. This figure appears in color at www.ajtmh.org.

**Figure 3.**
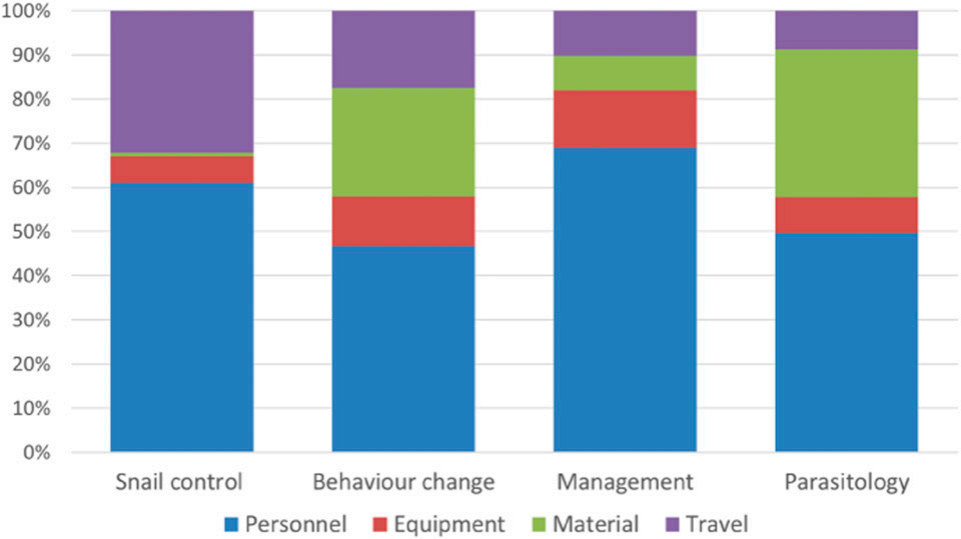
Cost structure by activities—total costs from 2012 to 2017 in Unguja. This figure appears in color at www.ajtmh.org.

The total estimated financial costs for implementing snail control activities over 5 years, excluding the costs for Bayluscide, were US$55,797 on Pemba (Table 1) and US$73,582 on Unguja (Table 2). The estimated cost per shehia covered was lower in Pemba (US$620 per shehia) than in Unguja (US$818 per shehia). This difference was larger in terms of cost per day in the field (US$96 per day on Pemba versus US$193 per day on Unguja) (Figure 1). The cost per shehia covered of the whole ZEST project on the two islands taken together was US$718. On both islands, a large part of the total costs was accounted by personnel (69% on Pemba and 61% on Unguja), followed by travel expenses (20% on Pemba and 32% on Unguja), as shown in Tables 1 and 2, Figures 2 and 3.

**Table 1:** Financial costs of the Zanzibar Elimination of Schistosomiasis Transmission project implemented in Pemba from 2011/2012 to 2017

Intervention cost category	Mass drug administration		Snail control		Behavior change		Management		Parasitologic survey	
	US$	%	US$	%	US$	%	US$	%	US$	%
Personnel	390,796.6	80.6	38,372.7	68.8	51,507.0	47.2	54,258.8	65.5	40,837.6	36.4
Equipment	13,070.2	2.7	3,389.4	6.1	10,398.0	9.5	9,657.6	11.7	5,598.5	5.0
Material	18,451.1	3.8	2,941.2	5.3	23,012.2	21.1	2,772.9	3.3	58,830.6	52.5
Travel	45,238.1	9.3	11,093.4	19.9	24,248.6	22.2	16,141.5	19.5	6,844.3	6.1
Other	17,331.6	3.6	–	–	–	–	–	–	–	–
Total costs	484,888	100	55,797	100	109,166	100	82,831	100	112,111	100

**Table 2:** Financial costs of the Zanzibar Elimination of Schistosomiasis Transmission project implemented in Unguja from 2011/2012 to 2017

Intervention cost category	Snail control		Behavior change		Management		Parasitologic survey	
	US$	%	US$	%	US$	%	US$	%
Personnel	44,828.0	60.9	72,785.0	46.7	142,919.0	69.1	82,580.0	49.6
Equipment	4,541.0	6.2	17,608.0	11.3	26,688.0	12.9	13,748.0	8.3
Material	495.0	0.7	38,187.0	24.5	16,157.0	7.8	55,727.0	33.4
Travel	23,717.6	32.2	27,248.7	17.5	21,174.0	10.2	14,544.7	8.7
Total costs	73,582	100.0	155,829	100.0	206,938	100.0	166,599	100.0

### Costs of behavior change interventions.

**Figure 4.**
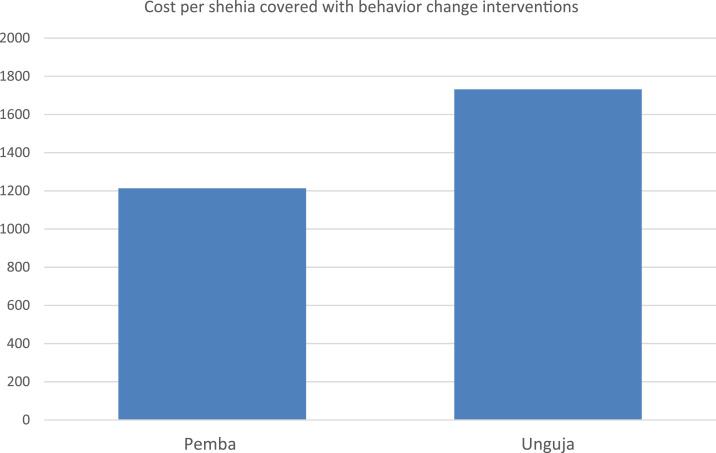
Behavior change: cost per shehia covered—inUS$. This figure appears in color at www.ajtmh.org.

The total estimated financial costs for implementing behavior change activities over 5 years were US$109,166 in Pemba (Table 1) and US$155,829 in Unguja (Table 2). The estimated cost per shehia was lower in Pemba (US$1,213) than in Unguja (US$1,731). On average, the costs per shehia were US$1,472 (Figure 4). On Pemba, the cost of personnel accounted for 47% of the total costs, whereas on Unguja, it accounted for 69%. The cost of equipment was higher on Unguja than on Pemba (Figures 2 and 3). On both islands, slightly more than 40% of the total costs was accounted by material and travel expenses.

### Costs of MDA.

Cost estimates for MDA were only available for Pemba and do not include the costs of donated praziquantel. The costs refer to the estimated 2.4 million treatments provided on Pemba over 4 years within eight rounds of MDA. The total cost of MDA amounted to US$484,887. The average cost for a single treatment was US$0.20 (Table 1). More than 80% of the total cost was accounted by personnel, followed by travel expenses (9.3%), material (3.8%), and equipment (2.7%) (Figure 1).

### Costs of parasitologic surveys.

The total costs of six parasitologic surveys were estimated to be US$112,110 on Pemba (Table 1) and US$166,599 on Unguja (Table 2). On average, the cost per survey was around US$18,685 on Pemba and US$27,776 on Unguja. The cost of personnel accounted for 36% of the total cost on Pemba and for almost 50% on Unguja (Figures 1 and 2). The travel expenses were higher on Unguja (US$14,544) than on Pemba (US$6,844), whereas the costs for materials were similar on both islands. In Unguja, the costs for laboratory equipment were much higher than in Pemba (US$13,748 versus US$5,598).

### Project management costs.

The estimated total costs of managing the ZEST project over the 5 years (from 2011/2012 to 2017) were US$82,831 on Pemba (Table 1) and US$206,938 on Unguja (Table 2). On average, the annual cost for managing the ZEST project was US$13,805 on Pemba and US$34,489 on Unguja.

## DISCUSSION

We estimated the financial costs of the ZEST project, analyzing programmatic and financial data made available by the project partners. The ultimate goal of the ZEST project was to eliminate urogenital schistosomiasis as a public health problem on the two islands of Zanzibar and to interrupt transmission of the disease in the longer run.^9,20^ Despite the limitations of using administrative data for estimating the costs of global health interventions,^28^ the findings of this study are relevant not only for schistosomiasis control and elimination efforts on Zanzibar but also for other NTD control programs elsewhere in sub-Saharan Africa.

First, the financial cost estimates include all the activities implemented by the various ZEST partners and stakeholders. Specifically, costs of the different interventions (i.e., MDA, snail control, and behavioral change activities), large-scale repeated cross-sectional parasitologic surveys that are necessary to assess the impact of interventions, and for managing the project were included. Such a comprehensive approach is rarely pursued in costing studies of global health interventions. Indeed, prior studies mainly estimated the costs of a single intervention or, in case of a package of intervention, did not provide disaggregated estimates by interventions, and lacked inclusion of ancillary but crucial activities such as those related to project management and monitoring.^5,8,28^ Our findings indicate, for example, the considerable costs related to project management activities. In fact, the estimated costs of managing the whole ZEST project were slightly higher than the estimated costs of behavioral change activities. It should be noted that managing a large-scale schistosomiasis elimination project is a complex undertaking that requires the availability of well-trained personnel. In addition, the estimated costs of the cross-sectional parasitologic surveys to assess longitudinally the impact of the interventions were relatively high. These findings point to the importance of considering the costs of monitoring activities and surveillance systems in the context^29,30^ of NTD elimination strategies. There are only a few studies that estimated the costs of surveillance systems, and most of them only provided aggregated cost estimates.^5,8,30–34^

Second, this study estimated the costs of the ZEST project disaggregating them according to how the interventions were geographically distributed. Although the disaggregated cost estimates for Pemba and Unguja have limitations due to the predefined budgets both islands had for implementing the surveys and interventions, these disaggregated estimates can be useful for informing decisions on the resources required for future schistosomiasis control and elimination efforts. In the ZEST project, the costs per shehia covered for snail control and for behavior change activities were higher on Unguja than on Pemba. The differences were mainly driven by the cost of transport and personnel. In Unguja, more people and at a higher level (principal investigator of research study and NTD program manager) were involved in managing the ZEST project, which included both research and programmatic parts. Taken together, the cost of personnel was 2.6-fold higher in Unguja than on Pemba.

Third, the cost estimates disaggregated by cost category provide important information of the relative importance of the different types of costs and related activities. Consistent with the results of previous studies,^5,29,35,36^ around 80% of the non–medicine-related costs of MDA was accounted by personnel. Similarly, personnel emerged as a major cost driver for snail control and behavior change interventions. Our findings further revealed that for snail control and behavior change interventions, travel costs were considerable and, for the behavior intervention, budgeting for consumable goods (e.g., health education activities and washing platforms), was important. In addition, the project management cost estimates show that besides personnel, to manage the implementation of schistosomiasis control interventions, a substantial budget to cover travel expenses is required.

Our findings should be interpreted with caution, as the study has several limitations. First, the costs estimates are based on a retrospective analysis of administrative data made available by the various ZEST partners. Hence, the analysis required considerable efforts to identify various resources used and reclassification of budget data to be attributed to the specific interventions and activities. Although this exercise proved to be feasible, we cannot guarantee that all reclassifications were accurate. This may be particularly relevant to the estimates of the project management costs allocated to the two islands. Second, using administrative data of a project supported by external partners may lead to cost estimates that are affected by budgeting rules and practice, and budgets predefined by external funders. Third, the costs presented in this study should be considered as conservative estimates, as they do not include support from external experts who nevertheless played a crucial role in the design, implementation, and longitudinal monitoring of the project. For example, the costs of behavior change interventions presented here do not include the costs of the consultant, who was crucial to develop the behavior change interventions and to train the staff of the behavior change team for implementation of interventions, and hence need to be interpreted with care. The cost estimates presented here include only financial costs and, hence, do not consider the costs of infrastructure that was already in place. In addition, the costs of MDA did not include the cost of praziquantel and the costs of snail control did not include the cost of Bayluscide. Both products were donated, and they therefore did not generate any financial expense. Praziquantel was donated to the ZNTD by the WHO, whereas Bayluscide was donated by Bayer Crop Sciences. Including these costs was beyond the scope of this study. Moreover, we consider the estimation of the economic value of donated products prone to methodologic challenges and uncertainties. Yet, it is worth noting that a recent literature review^8^ estimated the costs of praziquantel used in schistosomiasis control programs for MDA to represent a large part of the total economic cost of the intervention.

Despite these limitations, our study showed the value of exploiting administrative data to estimate costs of a package of global health interventions. The granular cost estimates presented here could, for example, be used by program managers to estimate the financial costs of scaling up a control program in Zanzibar or similar settings. Our study strengthens the evidence base of the financial costs and cost drivers of implementing a comprehensive package of schistosomiasis control and elimination interventions in different socio-ecological settings that can be used to inform decisions regarding the feasibility and affordability of schistosomiasis elimination strategies.

## Supplemental material

Supplemental materials

## References

[1] Lo NC Bogoch II Blackburn BG Raso G N’Goran EK Coulibaly JT Becker SL Abrams HB Utzinger J Andrews JR, 2015 Comparison of community-wide, integrated mass drug administration strategies for schistosomiasis and soil-transmitted helminthiasis: a cost-effectiveness modelling study. Lancet Glob Health 3: e629–e638. 26385302 10.1016/S2214-109X(15)00047-9

[2] Turner HC Walker M French MD Blake IM Churcher TS Basanez MG, 2014 Neglected tools for neglected diseases: mathematical models in economic evaluations. Trends Parasitol 30: 562–570. 25455565 10.1016/j.pt.2014.10.001

[3] Turner H French M Montresor A King C Rollinson D Toor J, 2020 Economic evaluations of human schistosomiasis interventions: a systematic review and identification of associated research needs [version 1; peer review: awaiting peer review]. Wellcome Open Res 5: 45. 32587899 10.12688/wellcomeopenres.15754.1 PMC7308887

[4] Tediosi F Steinmann P de Savigny D Tanner M, 2013 Developing eradication investment cases for onchocerciasis, lymphatic filariasis, and human African trypanosomiasis: rationale and main challenges. PLoS Negl Trop Dis 7: e2446. 24244762 10.1371/journal.pntd.0002446 PMC3820723

[5] Turner HC Truscott JE Hollingsworth TD Bettis AA Brooker SJ Anderson RM, 2015 Cost and cost-effectiveness of soil-transmitted helminth treatment programmes: systematic review and research needs. Parasit Vectors 8: 355. 26137945 10.1186/s13071-015-0885-3 PMC4499443

[6] Cunnama L 2016 Using top-down and bottom-up costing approaches in LMICs: the case for using both to assess the incremental costs of new technologies at scale. Health Econ 25 (Suppl 1): 53–66. 26763594 10.1002/hec.3295 PMC5066665

[7] Wang H Aas E Roman E Smith A, 2015 Comparison of different costing methods. Value Health 18: A687.

[8] Salari P Fürst T Knopp S Utzinger J Tediosi F, 2020 Cost of interventions to control schistosomiasis: a systematic review of the literature. PLoS Negl Trop Dis 14: e0008098. 32226008 10.1371/journal.pntd.0008098 PMC7145200

[9] Knopp S 2019 Evaluation of integrated interventions layered on mass drug administration for urogenital schistosomiasis elimination: a cluster-randomised trial. Lancet Glob Health 7: e1118–e1129. 31255591 10.1016/S2214-109X(19)30189-5 PMC6624424

[10] Knopp S 2019 A 5-year intervention study on elimination of urogenital schistosomiasis in Zanzibar: parasitological results of annual cross-sectional surveys. PLoS Negl Trop Dis 13: e0007268. 31059495 10.1371/journal.pntd.0007268 PMC6502312

[11] OCGS, 2019 Zanzibar Statistical Abstract 2018. Zanzibar, United Republic of Tanzania: Office of the Chief Government Statistician.

[12] Mc Cullough FS Krafft JH, 1975 Schistosomiasis in Zanzibar and Pemba. Report on a Mission 1 April–7 June 1975 Geneva, Switzerland: World Health Organization.

[13] Mgeni AF Kisumku UM McCullough FS Dixon H Yoon SS Mott KE, 1990 Metrifonate in the control of urinary schistosomiasis in Zanzibar. Bull World Health Organ 68: 721–730. 2127381 PMC2393165

[14] Savioli L Dixon H Kisumku UM Mott KE, 1989 Control of morbidity due to *Schistosoma haematobium* on Pemba Island: programme organization and management. Trop Med Parasitol 40: 189–194. 2505381

[15] Savioli L Hatz C Dixon H Kisumku UM Mott KE, 1990 Control of morbidity due to *Schistosoma haematobium* on Pemba Island: egg excretion and hematuria as indicators of infection. Am J Trop Med Hyg 43: 289–295. 2121056 10.4269/ajtmh.1990.43.289

[16] Goatly KD Jordan P, 1965 Schistosomiasis in Zanzibar and Pemba. East Afr Med J 42: 1–9. 14271180

[17] Guidi A Andolina C Makame Ame S Albonico M Cioli D Juma Haji H, 2010 Praziquantel efficacy and long-term appraisal of schistosomiasis control in Pemba Island. Trop Med Int Health 15: 614–618. 20214757 10.1111/j.1365-3156.2010.02488.x

[18] Knopp S Stothard JR Rollinson D Mohammed KA Khamis IS Marti H Utzinger J, 2013 From morbidity control to transmission control: time to change tactics against helminths on Unguja Island, Zanzibar. Acta Trop 128: 412–422. 21586268 10.1016/j.actatropica.2011.04.010

[19] Stothard JR French MD Khamis IS Basáñez MG Rollinson D, 2009 The epidemiology and control of urinary schistosomiasis and soil-transmitted helminthiasis in schoolchildren on Unguja island, Zanzibar. Trans R Soc Trop Med Hyg 103: 1031–1044. 19409588 10.1016/j.trstmh.2009.03.024

[20] Knopp S 2012 Study and implementation of urogenital schistosomiasis elimination in Zanzibar (Unguja and Pemba Islands) using an integrated multidisciplinary approach. BMC Public Health 12: 930. 23110494 10.1186/1471-2458-12-930 PMC3533998

[21] Campbell CH 2020 SCORE operational research on moving toward interruption of schistosomiasis transmission. Am J Trop Med Hyg 103: 58–65. 32400354 10.4269/ajtmh.19-0825 PMC7351301

[22] Allan F 2020 Snail-related contributions from the schistosomiasis consortium for operational research and evaluation program including xenomonitoring, focal mollusciciding, biological control, and modeling. Am J Trop Med Hyg 103: 66–79. 10.4269/ajtmh.19-0831 PMC7351297 32400353

[23] Celone M Person B Ali SM Lyimo JH Mohammed UA Khamis AN Mohammed YS Mohammed KA Rollinson D Knopp S, 2016 Increasing the reach: involving local muslim religious teachers in a behavioral intervention to eliminate urogenital schistosomiasis in Zanzibar. Acta Trop 163: 142–148. 27498244 10.1016/j.actatropica.2016.08.004 PMC5019290

[24] Person B Ali SM A’Kadir FM Ali JN Mohammed UA Mohammed KA Rollinson D Knopp S, 2016 Community knowledge, perceptions, and practices associated with urogenital schistosomiasis among school-aged children in Zanzibar, United Republic of Tanzania. PLoS Negl Trop Dis 10: e0004814. 27399310 10.1371/journal.pntd.0004814 PMC4939940

[25] Person B Knopp S Ali SM A’Kadir FM Khamis AN Ali JN Lymo JH Mohammed KA Rollinson D, 2016 Community co-designed schistosomiasis control interventions for school-aged children in Zanzibar. J Biosoc Sci 48 (Suppl 1): S56–S73. 27428066 10.1017/S0021932016000067

[26] GSA, 2019 Available at: https://www.eliminateschisto.org/resources/teacher-toolkit-for-intestinal-schistosomiasis. Accessed August 5, 2020.

[27] Knopp S 2013 Elimination of schistosomiasis transmission in Zanzibar: baseline findings before the onset of a randomized intervention trial. PLoS Negl Trop Dis 7: e2474. 24147165 10.1371/journal.pntd.0002474 PMC3798599

[28] Vassall A 2017 Reference Case for Estimating the Costs of Global Health Services and Interventions. Global Health Cost Consortium.

[29] Brooker S Kabatereine NB Fleming F Devlin N, 2008 Cost and cost-effectiveness of nationwide school-based helminth control in Uganda: intra-country variation and effects of scaling-up. Health Policy Plan 23: 24–35. 18024966 10.1093/heapol/czm041 PMC2637386

[30] Lee BY Bartsch SM Gorham KM, 2015 Chapter eight - economic and financial evaluation of neglected tropical diseases. Anderson RM Basáñez MG, eds. Advances in Parasitology. Cambridge, MA: Academic Press, 329–417. 10.1016/bs.apar.2015.01.002 25765199

[31] Kastner RJ Sicuri E Stone CM Matwale G Onapa A Tediosi F, 2017 How much will it cost to eradicate lymphatic filariasis? An analysis of the financial and economic costs of intensified efforts against lymphatic filariasis. PLoS Negl Trop Dis 11: e0005934. 28949987 10.1371/journal.pntd.0005934 PMC5630187

[32] Kim YE Sicuri E Tediosi F, 2015 Financial and economic costs of the elimination and eradication of onchocerciasis (river blindness) in Africa. PLoS Negl Trop Dis 9: e0004056. 26360917 10.1371/journal.pntd.0004056 PMC4567329

[33] Lenk EJ 2018 Socioeconomic benefit to individuals of achieving 2020 targets for four neglected tropical diseases controlled/eliminated by innovative and intensified disease management: human African trypanosomiasis, leprosy, visceral leishmaniasis, Chagas disease. PLoS Negl Trop Dis 12: e0006250. 29534061 10.1371/journal.pntd.0006250 PMC5849290

[34] Redekop WK 2017 The socioeconomic benefit to individuals of achieving the 2020 targets for five preventive chemotherapy neglected tropical diseases. PLoS Negl Trop Dis 11: e0005289. 28103243 10.1371/journal.pntd.0005289 PMC5313231

[35] Evans D McFarland D Adamani W Eigege A Miri E Schulz J Pede E Umbugadu C Ogbu-Pearse P Richards FO, 2011 Cost-effectiveness of triple drug administration (TDA) with praziquantel, ivermectin and albendazole for the prevention of neglected tropical diseases in Nigeria. Ann Trop Med Parasitol 105: 537–547. 22325813 10.1179/2047773211Y.0000000010 PMC4089800

[36] Leslie J Garba A Oliva EB Barkire A Tinni AA Djibo A Mounkaila I Fenwick A, 2011 Schistosomiasis and soil-transmitted helminth control in Niger: cost effectiveness of school based and community distributed mass drug administration [corrected]. PLoS Negl Trop Dis 5: e1326. 22022622 10.1371/journal.pntd.0001326 PMC3191121

